# Preference and Motivation Tests for Body Tactile Stimulation in Fish

**DOI:** 10.3390/ani11072042

**Published:** 2021-07-08

**Authors:** Ana Carolina dos Santos Gauy, Marcela Cesar Bolognesi, Guilherme Delgado Martins, Eliane Gonçalves-de-Freitas

**Affiliations:** 1 Departamento de Ciências Biológicas, Instituto de Biociências, Letras e Ciências Exatas, Universidade Estadual Paulista (UNESP), Rua Cristóvão Colombo, 2265, São José do Rio Preto 15054-000, SP, Brazil; ana.gauy@unesp.br (A.C.d.S.G.); marcela.bolognesi@unesp.br (M.C.B.); gui_hernan@yahoo.com.br (G.D.M.); 2 CAUNESP—Centro de Aquicultura da UNESP, Jaboticabal 14884-900, SP, Brazil

**Keywords:** choice, motivation, social stress, aggressive behavior, welfare

## Abstract

**Simple Summary:**

Body tactile stimulation, such as human massage therapy, is a way to relieve stress in humans and other animals, therefore it could improve animal health and welfare. This physical stimulation can also be done through artificial devices, as a sensory enrichment. However, before using it in an artificial environment, it is imperative to test whether animals perceive such enrichment as positive (searching for it spontaneously) or negative (avoiding it). Here, we tested whether the Nile tilapia fish search for or avoid tactile stimulation. We used a rectangular PVC frame, filled with vertical plastic sticks sided with silicone bristles that provided tactile stimulation when fish passed through them. We carried out preference and motivation tests, in which fish could choose to cross through the device with and without tactile stimulus. The same procedure was repeated after fish were exposed to either isolation or social stress. We found that fish crossed less by tactile device than by open areas. However, as fish spontaneously crossed through the bristles, and overcame an aversive high-intensity lighted route to reach the device, we conclude that tactile stimulation is not a negative condition. Thus, further studies can be designed to test several effects of tactile stimulation on the welfare of fish.

**Abstract:**

We tested whether territorial fish (Nile tilapia) perceive body tactile stimulation as a positive or negative resource. Individual male fish were placed for eight days in an aquarium containing a rectangular PVC frame, which was filled with vertical plastic sticks sided with silicone bristles in the middle of the tank. Fish passing this device received a tactile stimulus. The fish then underwent a preference test by choosing between areas half-with and half-without tactile bristles. Then, fish were submitted to a motivation test where they had to pass an aversive stimulus (bright light) to access the device. Fish were, then, paired to settle social rank, which occurs by way of fights (social stressor), and were assigned again to preference and motivation tests. A group without social stress was used as a control. Contrary to our expectations, fish preferred the area without tactile bristles, although subordinate fish reached tactile stimulation more than the dominant one. Social stress did not affect the preference and motivation, suggesting that fish do not perceive tactile stimulation as a stressor reliever. However, as fish did not avoid the stimulation, reached the device spontaneously, and faced an aversive stimulus to access it, we conclude that tactile stimulation is not a negative condition and, therefore, can be used in further studies regarding fish welfare.

## 1. Introduction

Animal welfare has been a frequent concern of human society in recent years [1]. Studies on fish welfare, for instance, have been increasing considerably [2] where fish are considered sentient beings [3], capable of conscious feelings and sensations [4]. In this scenario, although it represents a growing area of study, a common definition of welfare is not available since the emphasis on different aspects of animal biology can vary among schools [5]. The most traditional approach to evaluate fish welfare, for example, relies on indicators of animal function and physiological responses, such as catecholamine and corticosteroid levels in blood plasma. These hormones indicate stress levels variation [6], meaning that the higher the stress level, the poorer the welfare. Behavioral responses, such as the presence of abnormal behaviors [6], stereotyped behaviors [7], changes in aggressiveness [8] and cognitive performance [9], are also among the classic indicators. However, emotions and other feelings are more difficult to access in most non-human animals. Thus, one way to access these emotional states, as well as other variables that cannot be directly measured by physiological indicators, is the response of the animal from its perspective, regarding what is negative or positive for it.

Here, we will follow an approach that emerged from the latter statement, and which considers that the welfare of fish is good if they are healthy and are under conditions that were freely chosen [10,11]. Therefore, the perception of animal needs and what they want [12,13,14,15,16] can be achieved by preference tests [3,11,17,18]. These tests are designed to analyze how the animal feels by way of functional and behavioral traits [12] and the animal’s ability to choose the absence of negative states [19,20] and the presence of positive ones [21]. For example, the preference for a structured environment instead of barren ones in zebrafish (*Danio rerio*) and checker barbs (*Puntius oligolepis*) [22] or the preference for ground substrate in Mozambique tilapia [23] and Nile tilapia [24,25] show that such resources are imperative for keeping elevated welfare for these species. It is important to emphasize that choice and preference may represent different concepts depending on the context [18].

Preference can be complemented by motivation tests, which indicate how interested animals are in a particular resource or condition in the environment [16,17,19,26,27]. The rationale of this type of test is that, the more important a resource is to the animal, the higher the “price it is willing to pay” to reach it [13,15,28]. Motivation tests can, therefore, indicate how valuable a resource is to the animal [18], establishing a connection across perception, needs, and preference in the analysis of animal welfare [29]. In this framework, one of the most suitable experimental designs for studying motivation is the use of aversive obstacles or stimuli, which the animal must overcome to access a particular resource [17,27,29]. Physical obstacles, such as push-door operant devices, are commonly used [15,30]. In this model, costs are increased by adding weights to a door, which individuals must push to reach a resource. Psychological effort tests are also efficient to demonstrate animal motivation [27], for example, by creating pathways with an aversive stimulus that animals must surpass. These stimuli can be a route illuminated with a high-intensity light [27] or the presence of artificial water currents for fish [31]. Thus, an animal’s perception and desire for resources or attributes in the environment can be tested by preference and motivation approaches.

Body tactile stimulation is a way to improve welfare and increase positive state perception in vertebrates [32]. In mammals, for instance, touches can reduce stress [33,34,35]; massage therapy can relieve pain [36,37] and elevate serotonin levels in humans [33]; gentle stimulation with fingers on the dorsal surface of rats can minimize behavioral traits of anxiety and depression [38,39] and prevent learning deficits [40]. Tactile stimulation is also a type of sensory enrichment, which can improve welfare by enhancing cognition and adaptability to novel situations such as an artificial environment [41]. Recently, teleost fish have been tested for this effect as well. In an elegant study, Soares et al. [42] showed that body tactile stimulation reduces stress in client-cleaner fish (*Ctenochaetus striatus* and *Labroides dimidiatus*, respectively). These authors observed that the clients seek a tactile stimulus from a cleaner fish model and such stimulation reduces the cortisol levels of the client fish after confinement stress. Body tactile stimulation also reduces aggressive interaction in the territorial fish Nile tilapia [43], although it does not reduce cortisol levels. In this study, a tactile device was placed in the across the middle of the aquarium; then, the fish has to pass through it when swimming from one side of the aquarium to the other, without any chance of avoiding it [43]. Furthermore, client-cleaner tactile stimulation is a natural behavior of *Ctenochaetus striatus* and *Labroides dimidiatus* [42], while Nile tilapia males establish a rank-based hierarchy by aggressive interaction [44,45,46], a stressful type of body contact. Then, although tactile stimulation can reduce aggressive interaction in Nile tilapia [43], it is not yet clear if tactile stimulation has a positive or negative valence for this species. In this context, we used preference and motivation approaches to solve this problem. In the present study, we tested whether Nile tilapia spontaneously choose tactile stimulation and their motivation to access this stimulus.

Choices in the environment can be influenced by the emotional state of animals and the valence of the stimulus, that is, negative vs. positive and pleasant vs. unpleasant [47]. In this context, the social environment can affect the appraisal of a stimulus in fish [48]. For example, after an aggressive contest that leads to social status settling, social stress emerges, changing the internal state by stimulating the Hypothalamus-Pituitary-Interrenal axis and increasing cortisol levels [49]. This change in internal state can change the mood and the appraisal of a stimulus in the environment [47]. If the tactile stimulation is perceived as a positive valence, as it is for other vertebrates, we expect that the fish would choose to access that physical stimulus on its own, would not avoid it, and would be more motivated to access tactile stimulation to counteract the negative state from aggressive interaction, that is, social stress. Nile tilapia, *Oreochromis niloticus* (L.), is the third most produced species in aquaculture [50], and, as a cichlid fish, has been widely used as a model for physiology and behavioral research [32]. Although the literature provides a lot of knowledge regarding Nile tilapia physiology and behavior, a good scenario regarding welfare is still incipient. The FishEthoBase Project [51], for example, shows that this species is still under the optimum welfare score, and the knowledge about welfare indicators is still rudimentary [52]. Therefore, understanding the tools that improve the welfare of these animals is highly relevant [8,32,53,54].

## 2. Methods

### 2.1. Fish Housing

Adult males of GIFT (Genetically Improved Farmed Tilapia) Nile tilapia, from a commercial supplier, were kept in outdoor tanks at the university. The fish were taken to the laboratory, where they were acclimated for 15 days in a polypropylene tank (ca. 500 L, 1 fish/10 L) with water at 28 °C and a 12L:12D light regime (7:00 a.m. to 7:00 p.m.). The fish were fed with tropical fish food (28% crude protein, to apparent satiety) once a day. The water quality was maintained by biological filters (400 L/h) and constant aeration.

### 2.2. Tactile Stimulation

**Figure 1:**
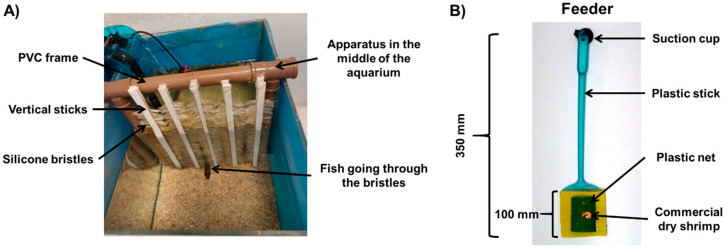
Tactile stimulation device. (**A**) Tactile stimulation device placed centrally in the aquarium with a fish going through the tactile bristles. (**B**) The feeder that was used to stimulate fish to go through the tactile device during the exposure phase.

Body tactile stimulation was provided by a rectangular PVC (polyvinyl chloride) frame, with vertical plastic sticks filled with silicone bristles on each side. The apparatus was placed in the middle of the aquarium so that fish received tactile stimulation when crossing through the bristled sticks (Figure 1A). To stimulate the fish to cross through the apparatus, we offered food (dry shrimps) attached to a feeder (Figure 1B) at the opposite side of the fish’s position in the aquarium.

In preliminary studies, we observed that fish took a long time to get to the food from the feeder. Thus, we provided a habituation period before experiments began. Fish from the 500-L tanks were, then, transferred to a glass aquarium (60 × 60 × 40 cm, ca. 140 L, four fish per aquarium), where they remained for 3 days under the same conditions as those used in acclimation, except for feeding. During this period, the animals were fed with dry shrimp attached to the feeder three times in the morning (8:00, 9:30, and 11:00 a.m.) and three times in the afternoon (2:00, 3:30, and 5:00 p.m.). By using this protocol, the fish quickly took food when the experiment with the tactile apparatus started.

### 2.3. Experimental Design

**Figure 2:**
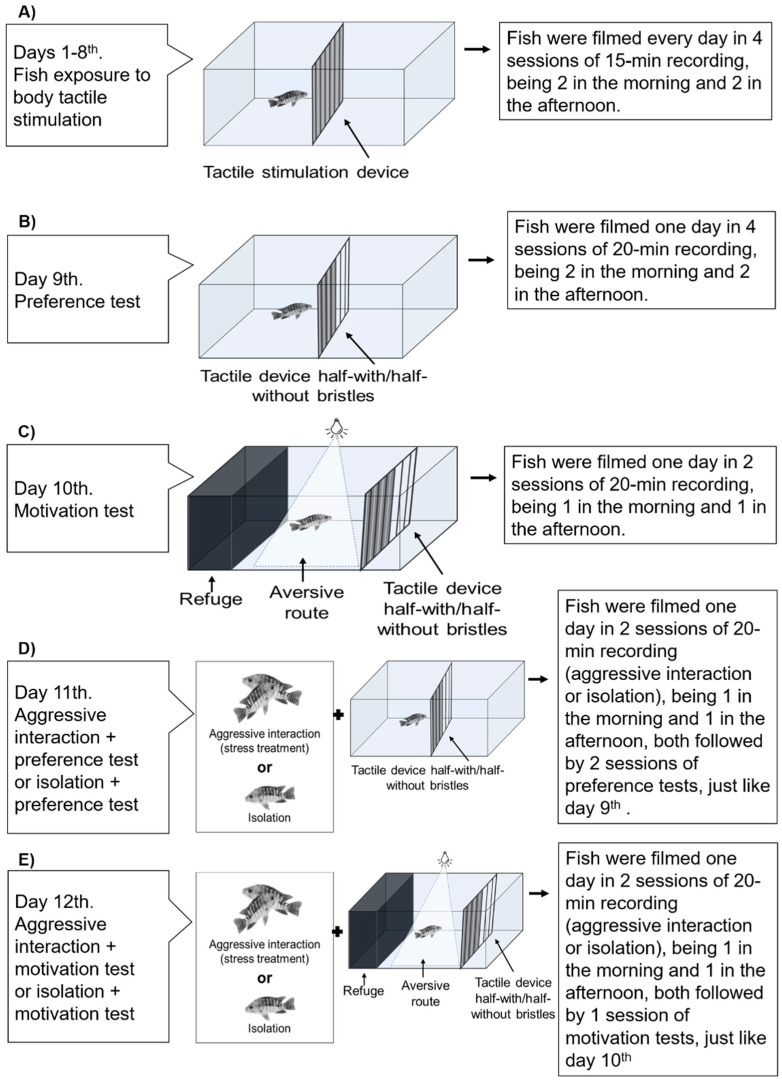
Protocol timeline. The steps of the experimental strategy showing the sequence of tests that fish were assigned to over 12 days. (**A**) Fish were isolated in glass aquaria and exposed to tactile stimulation for 8 days. (**B**) Preference test wherein half of the sticks in the apparatus had the bristles removed to allow spontaneous choices between tactile or no tactile stimulation. (**C**) Motivation test, in which fish had to leave a refuge (dark plastic cover) and surpass an aversive route (light path) to reach the tactile apparatus. (**D**) Fish were randomly paired and exposed to social stress (aggressive interaction) or isolation in another aquarium as a control for handling; they were, then, tested for preference, exactly as on the 9th day. (**E**) Fish were paired again as in the previous day and, then, tested for motivation, exactly as on the 10th day. The way animals were recorded is shown in each step.

The general strategy of the study consisted of a sequence of tests over 12 days. Firstly, fish were isolated and exposed to body tactile stimulation for 8 days and, over the next two days, they were tested with preference and motivation tests. Afterward, they were subjected to a stressful condition (social stress) and were tested again for preference and motivation in the remaining two days. A treatment in which fish were removed from the original aquarium and isolated in another one was used as the control for handling. Therefore, we tested two treatments, herein named “social stress treatment” and “isolation treatment” with *n* = 16 for each one. This design allowed us to analyze whether fish freely seek tactile stimulation and, then, whether stressful conditions increase the choice to seek the stimulus, that is, increase the valence of the stimulus. The protocol is summarized in Figure 2.

### 2.4. Exposure to Tactile Stimulation

Animals were isolated in a glass aquarium (80 × 30 × 40 cm, ca. 90 L) with the tactile apparatus in the center (Figure 2A). They were fed twice in the morning (9:00 and 11:00 a.m.) and the afternoon (2:00 and 4:00 p.m.) for 8 days. To test the exposure to the stimulus, fish were video recorded by cameras placed above the aquaria for 15 min, 5 min before, 5 min during and 5 min after feeding. For data analysis, we considered only the times before and after feeding (10 min) to avoid biases from the presence of food. This protocol had been previously tested and validated by Bolognesi et al. [43].

### 2.5. Preference Test

In this test, the tactile apparatus had half of its area filled with, and half without, the silicone bristles, so that fish could choose one of these areas (with or without tactile stimulation) (Figure 2B). To control for cues that could bias the animal’s preference, we covered all the aquarium’s walls with an opaque blue plastic and alternated the position of the area with or without bristles among the replicates to avoid the place effect.

Fish were video recorded (from above) in four daily sessions (20 min each recording), twice in the morning (9:00 and 11:00 a.m.) and twice in the afternoon (2:00 and 4:00 p.m.). The test was repeated four times to evaluate consistency by way of the Preference Index (see data analysis). We recorded the spontaneous choice of the fish without using the feeder, thus avoiding the effect of conditioning on the preference.

### 2.6. Motivation Test

The motivation test was based on the study of Maia et al. [27], to test the fish’s propensity to overcome an aversive obstacle to reach a resource. For this, an area in one aquarium’s end was covered with black plastic, creating a refuge. The tactile device was also made of half-with/half-without tactile bristles and was placed on the opposite side of the refuge (Figure 2C). A bright light is an aversive stimulus for several fish species [55,56], including Nile tilapia [57]. Thus, we placed an LED lamp (900 lumens) 20 cm above the aquarium’s surface between the refuge and the apparatus to create an aversive route for the animal. The motivation was inferred from the latency for leaving the refuge and swimming through the aversive route to access the tactile device. The animals remained in the refuge for 5 min and then, a frontal door was open, and fish were video recorded for 20 min. The latency to access the apparatus after leaving the refuge, as well as the number of crossings in both areas of the apparatus, were recorded.

### 2.7. Social Stress Test

Aggressive interaction is a high stressor for territorial fish [58,59]; therefore, we used fights as a proxy for social stress. Fish that were already tested for preference and motivation were withdrawn from their original aquaria and were randomly paired (20 min) with another individual, also from the experiment, in a neutral aquarium. The same individuals were paired twice in the day (8:30 a.m. and 1:30 p.m.) and were then tested again on the preference test exactly as previously tested, after both pairing sessions (Figure 2D). On the next day, they were assigned again to an aggressive interaction and to the motivation test, also in the same way as tested before the social stress (Figure 2E). Since handling increases stress in tilapias [60,61], we used a treatment in which fish were grabbed from their aquarium and were isolated in a neutral one as a control for manipulation.

We analyzed the aggressive interactions performed by each fish during the pairing according to the ethogram described for Nile tilapia [62]. Aggressive behavior was labeled as attacks and displays. Attacks are interactions with physical contact and higher energy expenditure (such as nipping, mouth fight and undulation), and displays are interactions without physical contact that have a lower energy expenditure [63] (such as the lateral threat and circling). To identify the fish’s social rank, we used a dominance index calculated as the relative frequency of individual aggressive acts over the number of aggressive acts given plus received for each fish [62]. This value varies from 0 to 1; the winning fish is the one with an index closer to 1 and, the loser is the fish that is rated closer to zero [62]. The association between aggressive interactions and stress level has already been well established for Nile tilapia [43,59,64] and therefore we did not measure the cortisol levels of the fish.

### 2.8. Experimental Details

Before isolation in the experimental aquarium, on the first day of the experiment, the animals were anesthetized by immersion in Benzocaine (0.03 g·L^−1^), weighted, sized, and individually identified by green elastomer (VIE tags) that were inserted under 2 or 3 scales on each side of the body. The mean (± S.E.) standard length and weight of the fish were, respectively: Social stress treatment: 10.76 cm ± 0.61 cm; 41.74 g ± 7.86 g; Isolation treatment: 10.42 cm ± 0.71 cm; 39.02 g ± 9.55 g. There were no significant differences between fish sizes (Unpaired *t*-test, *t*_(30)_ = 1.46, *p* = 0.15) and weight (*t*_(30)_ = 0.87, *p* = 0.38) between the treatments.

Fish were observed in glass aquaria covered with blue plastic to avoid visual contact with animals from neighboring aquaria, and because the blue color is less stressful for Nile tilapia [65]. Video recording was performed by cameras placed above the aquaria that send the records to a computer in an adjacent room, therefore avoiding the observer’s influence on fish behavior. Cameras placed on tripods in front of the aquaria were used to record aggressive interactions. Abiotic variables were controlled. The photoperiod was set to 12L:12D (from 7:00 a.m. to 7:00 p.m.) and the temperature to 28° C. The water quality was monitored with commercial kits (LabconTest, Petaluma, CA, USA) and electronic devices (Hanna HI98127, Smithfield, RI, USA): Ammonia (0.025 ppm) and Nitrite (0.125 ppm); pH (8.36 ± 0.25—representing the alkaline water from the well that feeds the lab).

### 2.9. Data Analysis

The data were tested for normality with the Kolmogorov–Smirnov test and homoscedasticity with Fmax [66]. When necessary, data were square-root transformed to reach homoscedasticity. The number of crossings in the initial 8 days of contact with the tactile apparatus was evaluated by ANOVA and a repeated measures mixed model ANOVA was used to compare between the treatments (social stress vs. isolation) and within periods before and after social stress or isolation; also, within the number of crossings (choices) by areas with or without tactile bristles in the preference and motivation tests. This test was also used to evaluate the latency to reach the tactile device between treatments and within periods before and after social stress or isolation. We also analyzed the responses of winner and loser fish regarding preference and motivation tests with the same statistical approach. Data were completed with the Newman–Keuls post hoc test. Data of interest were contrasted by Planned Comparisons. All data were analyzed with the software Statistica 13.4.

Besides these analyses, we also used a preference index (PI) based on the study of Maia and Volpato [18] to complement the analysis of the number of crossings in the preference test. The index calculation predicts that the most recent choices may better represent what the animal prefers considering its choices over time [18,27]. Therefore, it can be used to show consistency among choices. The Preference Index follows the steps exemplified in Table 1. Firstly, the frequency of crossings through each apparatus’ half (with or without tactile stimulation) was summed. Then, we calculated the areas above the line of cumulative frequency, which increase as the preference trials progress. The positive PIs represent the preferred individual option; meanwhile, the negative PI values indicate the non-preferred option, and the values of PI represent the intensity of preference/dispreference responses [18,27]. We calculated the PI values for each fish using the four test trials before, and the four test trials after, manipulation (social stress or isolation treatment).

## 3. Ethical Note

This study followed the Ethical Principles adopted by the National Council for the Control of Animal Experimentation (CONCEA/Brazil) and was approved by the Committee on Ethics in Animal Use, IBILCE, UNESP, São José do Rio Preto, permit #171/2017.

## 4. Results

### 4.1. Previous Exposure to Tactile Stimulation

**Figure 3:**
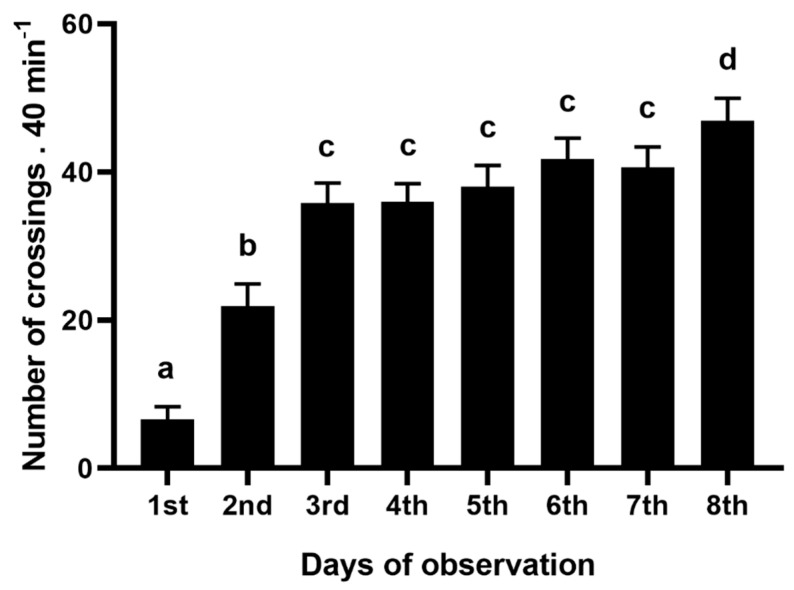
Prior exposure to tactile stimulation. Number of crossings through the tactile bristles during the eight days fish were exposed to the tactile device (*n* = 32). Data are mean ± SE. ANOVA for repeated measures followed by SNK post hoc test. Letters compare means between days. Similar letters indicate no significant differences.

Fish successfully crossed by the tactile apparatus in the eight initial days. The number of crossings was different among the observation sessions (F_(7, 217)_ = 50.29, *p* < 0.00001, Figure 3) and, according to the SNK post hoc test, it increased from the beginning (*p* < 0.0001) and stabilized after the third day (*p* > 0.15). Crossing increased on day eight, this session being higher than all other observations (*p* < 0.047).

### 4.2. Preference Test

**Figure 4:**
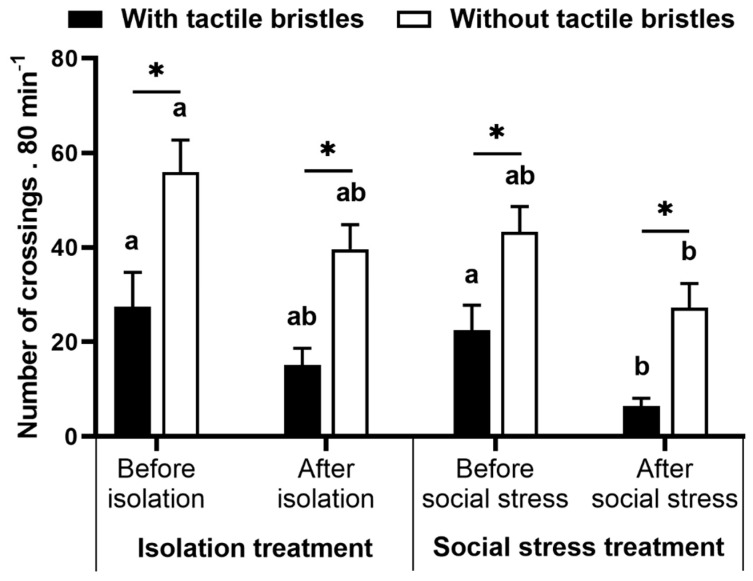
Preference test. Number of crossings through the area with or without tactile bristles over the periods before and after manipulation in isolation and social stress treatment. Data are mean ± SE. Mixed model ANOVA followed by SNK post hoc test. The asterisk indicates significant differences between areas with and without tactile bristles within treatments in the periods. Letters compare before and after manipulation for control and treatment groups. Similar letters indicate no significant differences.

For this analysis, we removed one replicate from the isolation treatment and two replicates from the social stress treatment because they were motionless and did not make any choice during the tests. We observed a significant effect of treatment on the number of crossings through the tactile apparatus in the preference test (with vs. without social stress; F_(1,27)_ = 5.18, *p* = 0.031, Figure 4), as well differences within choices (with vs. without bristles; F_(3,81)_ = 23.08, *p* < 0.0001). Before social stress or isolation, fish chose to cross more times by the half without tactile bristles both in social stress (*p* = 0.027) and isolation treatment (*p* = 0.002) groups. This pattern was maintained after manipulation (*p* = 0.002 and *p* = 0.004 for social stress and isolation treatment, respectively). Crossing by the tactile device before and after isolation was similar in the control group (*p* > 0.212). However, crossing by the half with bristles were reduced after social stress (*p* = 0.010) and were similar in the half without bristles (*p* = 0.085).

### 4.3. Preference Index

**Figure 5:**
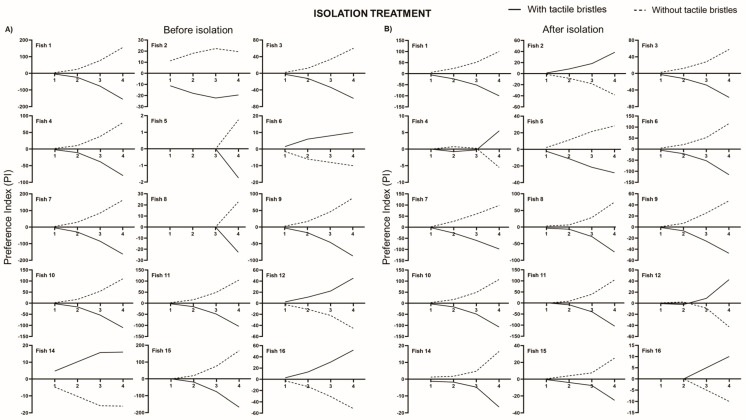

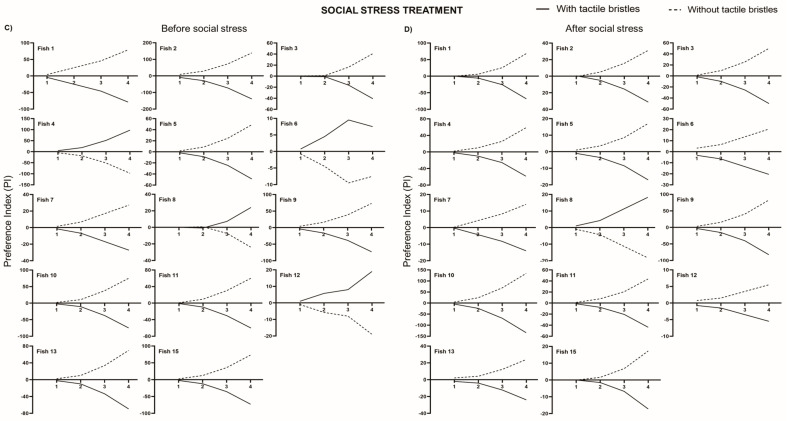
Preference index for the area with or without tactile bristles. Preference index of each fish from the isolation treatment (**A**) before and (**B**) after manipulation (isolation, *n* = 15); and from social stress treatment (**C**) before and (**D**) after manipulation (aggressive interaction, *n* = 14). We did not show fish 13 (isolation treatment), 14, and 16 (social stress treatment) in these analyses because they became motionless and did not cross through the tactile stimulator in one of the periods.

The preference index showed a consistent individual choice, following the tendency of the former analysis. In the isolation treatment, only four out of 15 fish preferred to cross by the area with bristles both before (Figure 5A) and after (Figure 5B) isolation, although the preference changed for two of them. In the social stress treatment, four out of 14 fish preferred to access the area with bristles before social stress (Figure 5C), but only one individual kept the preference for tactile stimulation after social stress (Figure 5D).

### 4.4. Motivation Test

**Figure 6:**
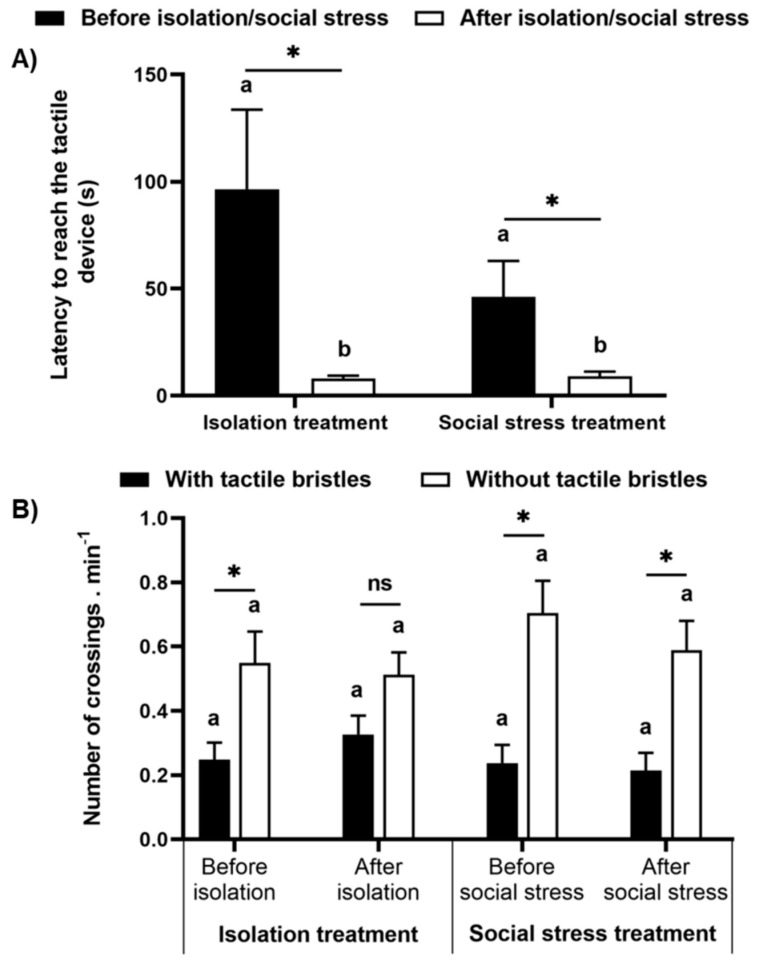
Motivation test. (**A**) Latency to access the tactile device and (**B**) the number of crossings per minute through the areas with and without tactile bristles after the fish leave the refuge, in both periods, before and after manipulation (isolation or aggressive interaction). Data are mean ± SE. Mixed model ANOVA followed by SNK post hoc test. The asterisk indicates significant differences between (**A**) periods before and after manipulation within treatments and (**B**) the areas with and without tactile bristles within treatments in the periods. Letters compare periods before and after manipulation (**A**) between treatments and (**B**) for control and treatment groups. Similar letters indicate no significant differences.

Fish were motivated to reach the tactile apparatus a similar amount between the treatments, and a preference for the area without tactile bristles after overcoming the light path was observed again. No difference was observed for the latency to reach the apparatus after leaving the refuge between the treatments (F_(1,27)_ = 0.081, *p* = 0.78), but was reduced after social stress and isolation (F_(1,27)_ = 0.1381, *p* = 0.0009, Figure 6A). As the latency to leave the dark area varied for each fish, we analyzed the number of crossings per minute after the fish left the refuge. We did not find a difference between the number of crossings for the isolation and social stress treatments (F_(1,23)_ = 0.042, *p* = 0.84, Figure 6B). However, we found a significant difference within treatment (F_(3,69)_ = 13.21, *p* < 0.00001). In the isolation treatment, fish crossing by the half without tactile bristles was marginally higher than by the area with tactile bristles (*p* = 0.058) and was similar between those areas after isolation (*p* = 0.08). In the social stress treatment, fish crossed more through the area without bristles both before and after social stress (*p* < 0.007, Figure 6B).

### 4.5. Social Rank

**Figure 7:**
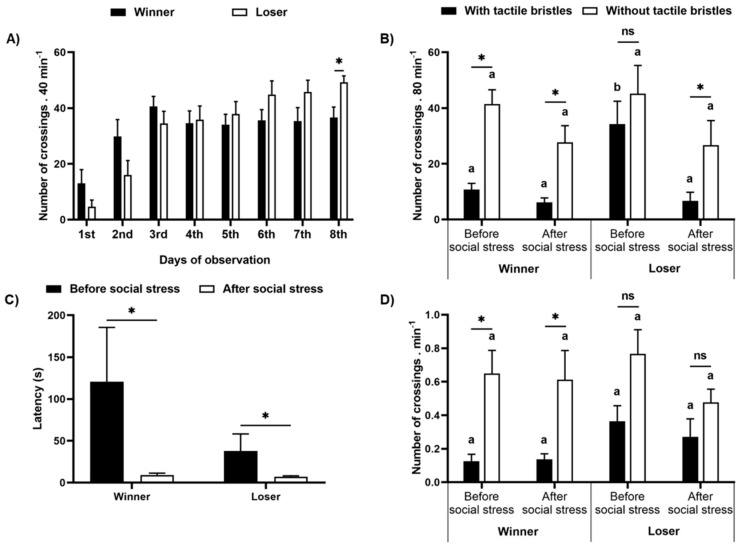
Winner and loser fish responses to preference and motivation tests. The number of crossings through the tactile bristles during the eight days fish were exposed to the tactile device (**A**); and the areas with and without bristles in the preference test, both before and after aggressive interaction (**B**). The latency to access the tactile device (**C**) and the number of crossing through the areas with or without tactile bristles (**D**) during the motivation test, both before and after the social stress, for winner and loser fish. Data are mean ± SE. Mixed model ANOVA followed by (**A**) Planned comparisons or (**B**–**D**) SNK post hoc test. The asterisk indicates significant differences between (**A**) ranks within days; (**B**,**D**) the areas with and without tactile bristles within ranks in the periods; (**C**) periods before and after stress within ranks. (**B**,**D**) letters compare periods before and after aggressive interaction for winner and loser fish. Similar letters indicate no significant differences.

We also look for possible differences between winner and loser fish in the treatment with social stress and analyzed the number of crossings through the tactile apparatus accordingly. There was a significant interaction between the social rank and the days of observation (F_(7,98)_ = 4.37, *p* = 0.0003, Figure 7A). By contrasting data with planned comparisons, we found that the losers’ crosses increased over time, so that it was marginally higher than the winners’ on day seven (F_(1,14)_ = 4.088, *p* = 0.066), and was significantly higher on day eight (F_(1,14)_ = 7.54, *p* = 0.016).

In the preference test, there was no difference between winner/loser fish (F_(1,12)_ = 0.96, *p* = 0.347), but we found differences between areas with and without tactile bristles within ranks (F_(3,36)_ = 17.00, *p* < 0.0001, Figure 7B). Winners crossed more frequently through the area without tactile bristles both before and after social stress (*p* < 0.012), whereas losers crossed similarly between the two areas before stress (*p* = 0.49) and increased crosses by the area without bristles after social stress (*p* = 0.018). The choices of loser fish to cross by the area with bristles were marginally higher than those of the winners before stress (*p* = 0.055) but were similar after stress (*p* = 0.88).

We did not observe an effect of winner/loser on the latency to reach the tactile device (F_(1,12)_ = 0.42, *p* = 0.53, Figure 7C). However, the latency reduced after social stress in both winner and loser fish (F_(1,12)_ = 5.28, *p* = 0.04). Winner/loser fish crossed similarly through the areas with or without bristles (F_(1,12)_ = 0.42, *p* = 0.53) but some differences were found within periods (F_(3,36)_ = 13.40, *p* = 0.000005, Figure 7D). Winner fish always chose the area without bristles both before and after social stress (*p* < 0.004). However, loser fish showed no preferences between these areas, both before and after social stress (*p* > 0.06).

## 5. Discussion

Preference and motivation tests are a way to understand what animals want and what they do not, therefore providing information regarding the positive or negative valence of stimuli according to the animal’s appraisal. Here, we showed for the first time that territorial fish do not avoid tactile stimulation even when there is a chance to avoid it. Nonetheless, contrary to our predictions, social stress did not increase the preference and motivation for tactile stimulation, showing, at first sight, that fish do not use tactile stimulation to relieve the effects of stress. We detected an effect of social rank, with loser fish accessing tactile stimulation more than winner fish. Thus, although fish chose to pass more frequently for the path without bristles, our results indicate that tactile stimulation is not aversive, therefore, it can be used in further studies regarding fish welfare.

The pattern of crossings in the initial eight days of the experiment indicates that the tactile apparatus was efficient for providing body tactile stimulation, as previously showed by Bolognesi et al. [43], thus validating our protocol. Fish gradually increased the crossings through the apparatus irrespective of food presentation, which means that fish need some time to adjust to the presence of the environment’s novelty, as the apparatus is a novel object [43]. A complete discussion of this device has already been provided by Bolognesi et al. [43]. Thus, the first part of this study succeeded in offering tactile exposure for fish.

When the access to an attribute that is not part of the natural range of an animal’s life increases, that resource probably has positive valence [13,15,47]. However, despite fish having spontaneously crossed through the tactile device in the first phase, they did not show a preference for this condition, as they chose to pass more frequently through the path free of silicone bristles. Natural physical contacts in Nile tilapia are mainly related to aggressive interactions [49], which is different to the contacts of the client–cleaner interactions in coral reef species [67], or any other non-aggressive contact, such as in shoal species [68]. In this way, the artificial contact provided by the tactile apparatus could be interpreted as a negative interaction by the fish. If so, we would expect animals to completely avoid tactile bristles, which was not the case. Another explanation would be the fact that the stimulator apparatus could act as a visual barrier to the fish. For cichlids, including Nile tilapia, vision plays a fundamental role in the species’ ecology [69,70] and is an important sensorial cue of movement and space localization [71], so fish would prefer to move by an area with no restriction to their visual perception of the environment. As physical barriers could hinder swimming [72], fish would avoid them. We consider the latter to be the most plausible explanation for our results, meaning that fish swim more frequently by the area free of bristles because it is an easier path, and they make a decision to go through a less easy area; otherwise, we would observe fish avoiding the area with bristles throughout the tests.

The preference indexes obtained for the preference test reinforce the general idea that most of the fish do not prefer to move through the tactile device. However, the PI also shows that the access to tactile stimulation varies individually, as shown for other resource types in Nile tilapia, such as environmental color [73], substrate type [25], and shelter [74]. Therefore, we must consider that these fish individually evaluate preferences, instead of quickly concluding that territorial fish do not prefer tactile stimulation.

Besides not avoiding stimulating bristles, the fish were motivated to cross an aversive route to access the tactile stimulator. It is possible that the route of high illumination did not represent a truly aversive stimulus for Nile tilapia. However, Nile tilapia are sensitive to increased light intensity [75], and the lumens used in this study have already been applied to other fish species in motivation tests [27]. The fact that the animals left a shaded area and then crossed from one side of an aquarium to another, where the apparatus was placed, indicates that fish take some risk to reach the tactile stimulation. Interestingly, fish took less time to cross by the light route after manipulation (isolation or social stress). We can interpret this as an increase in motivation to reach the device. On the other hand, because the result was similar between treatments, they probably learned that the route is not so risky. In fact, Nile tilapia show high demanding cognitive ability, which allows fish to cope with environmental challenges [76].

Bolognesi et al. [43] have already shown that tactile stimulation does not lower cortisol after social and non-social stress in Nile tilapia males. However, cortisol per se may not be the best indicator of a negative or positive effect of tactile stimulation. In this way, preference tests provide an advantage for evidencing animal appraisal, irrespective of association with physiological indicators [3,48]. Thus, considering the hypothesis that tactile stimulation would have a positive effect on relieving the consequences from a negative stimulus, we predicted that a social stressor would increase the access of the animals to tactile stimulation, as previously observed by Bolognesi et al. [43]. They found an increase in fish movement within the environment after socially aggressive interaction, leading the fish to pass through the tactile apparatus in the middle of the aquarium. However, we observed a reduction of crossings by the bristles and a tendency to reduce passing by the area without bristles in the preference test. This suggests that the social stressor in our study had reduced the overall activity of the fish, for instance, decreasing movement [77] and swimming performance [78,79], which is expected in stressed animals. However, we have no evidence of changes in the animals’ motivation to perform some spatial task [80,81].

Social isolation has been used as a control condition in several behavioral and physiological studies, despite being an important stressor for social fish [82]. In the present study, for instance, this is the only way to test for individual preferences and motivation. Thus, isolation was used in this study for understanding individuals’ motivation and preferences, as well as as a control for fish manipulation, since handling increases cortisol levels in Nile tilapia [60,61]. Moreover, social isolation is a lighter stressor than pairing in Nile tilapia, as the cortisol levels increase more after aggressive interaction than after isolation [59]. Therefore, we expected to find differences between treatments, such as a higher frequency of crossing through tactile bristles after social stress than after isolation. The reduced activity in both treatments, however, indicates that tactile stimulation does not act as a stress reliever irrespective of the intensity of the stressor. We found an association between the number of crossings in the eight days before preference and motivation testing with those animals that more frequently accessed the stimulation losing the contest. Tactile stimulation reduces the aggressiveness of Nile tilapia [43], therefore we speculate that individuals who were more exposed to tactile stimulation should become less aggressive and lose the contest, as aggressive ability defines the winning and losing individuals [8]. Another interesting fact about loser fish is that they accessed the areas with and without stimulation equally in the preference test before the stressor, as well as in the motivation test, unlike the winner fish. However, after the social stressor, the winner and loser fish showed a similar number of crossings by the stimulator in the preference test and a similar motivation to reach the device. It is known that subordinate and dominant Nile tilapia males could be similarly stressed when the social hierarchy is established [59]. On the other hand, the effects of agonistic interactions can be stronger for subordinate individuals over long periods [58], which could explain the reduced crossings through the area with and without tactile stimulation after the contests.

In this study, we observed that the pattern of preference for and motivation responses to tactile stimulation is very variable among individuals of Nile tilapia. Although we had tested only two possible choices, that is, with and without tactile stimulation, the fish did not avoid the tactile stimulus, meaning that it is not aversive (e.g., Maia et al. [74]). By analyzing preference and motivation together, we can extrapolate the individual views of each test, showing that even if an option is not accessed more often, animals may rather be motivated to access this option, and consequently, this condition may represent a positive valence from the perspective of the animal itself. We offer, then, an alternative interpretation on choice tests.

## 6. Conclusions

We conclude that tactile stimulation does not represent a negative valence, as animals do not avoid contact with the tactile bristles, even after a stressful stimulus. Furthermore, animals are motivated to access tactile stimulation even when they face an aversive stimulus in order to gain such access, suggesting that the tactile stimulation may instead represent a positive valence. Further studies can now be designed to test several effects of tactile stimulation on fish welfare, including to clarify the association between body tactile stimulation and social interaction.

## Figures and Tables

**Table 1:** Preference index steps. PI values of the fish one from social stress group going through the part of the device with tactile bristles before aggressive interaction during the 4 observations. Here, we are showing the fish 1, the same as Figure 5C, as an example. The PI indicates that fish did not prefer the tactile stimulation area. For details about calculations see Maia and Volpato [18].

Test Session	Row Frequency	Cumulative Frequency	Area	Cumulative Area	Expected Area	Area Variation	Preference Index (PI)
1	2	2	1	1	5.25	−4.25	−4.25
2	6	8	9	10	30.75	−20.75	−25
3	8	16	20	30	50.75	−20.75	−45.75
4	5	21	17.5	47.5	80.5	−33	−78.75

## Data Availability

The data presented in this study are available on request from the corresponding author.
